# A time-dependent predictive model for cardiocerebral vascular events in chronic hemodialysis patients: insights from a prospective study

**DOI:** 10.3389/fmed.2025.1481866

**Published:** 2025-06-04

**Authors:** Haowen Zhong, Mengbi Zhang, Yingye Xie, Yuqin Qin, Na Xie, Yuqiu Ye, Heng Li, Hongquan Peng, Xun Liu, Xiaoyan Su, Shaohong Li

**Affiliations:** ^1^Department of Nephrology, Dongguan Tungwah Hospital, Dongguan, China; ^2^Dongguan Key Laboratory of Precise Prevention & Treatment of Chronic Kidney Disease and Complications, Dongguan, China; ^3^KingPoint Data Technology Co., Ltd., Guangzhou, China; ^4^Department of Cardiology, Dongguan SongShan Lake Tungwah Hospital, Dongguan, China; ^5^Department of Nephrology, Kiang Wu Hospital, Macau, Macao SAR, China; ^6^Department of Nephrology, The Third Affiliated Hospital of Sun Yat-sen University, Guangzhou, China; ^7^Department of Emergency Medicine, Dongguan Tungwah Hospital, Dongguan, China

**Keywords:** time-dependent, predictive model, cardiocerebral vascular events, hemodialysis, laboratory tests

## Abstract

**Context:**

The conventional risk factors for cardiocerebral vascular events (CVCs) in non-Hemodialysis (HD) patients cannot be directly applied to HD patients due to the unique characteristics of this population. More accurate information on the risk of progression to CVCs is needed for clinical decisions.

**Objective:**

To develop and validate time-dependent predictive models for the progression of CVCs in HD patients.

**Design, setting, and participants:**

Development and validation of time-dependent predictive models using demographic, clinical, and laboratory data from 3 dialysis centers between 2017 and 2021. These models were developed using time-dependent Cox proportional hazards regression and assessed for discrimination using the concordance index, goodness of fit using the Akaike information criterion and net reclassification improvement.

**Main outcome measures:**

CVCs included acute heart failure, acute hematencephalon, cardiac or brain-derived death, acute myocardial infarction, acute cerebral infarction, ischemic cardiomyopathy, unstable angina pectoris, and stable angina pectoris.

**Results:**

The development and validation cohorts included 233 and 215 patients, respectively. The most accurate model included age, sex, hemoglobin, serum albumin, serum phosphate, white blood cell count, blood flow rate and ultrafiltration volume during HD (C index, 0.704; 95% CI, 0.639–0.768 in the development cohort and 0.775; 95% CI, 0.706–0.843 in the validation cohort). In the validation cohort, this model was more accurate than a model containing variables whose *p* value in the Cox proportional hazards regression was less than 0.05 (NRI: 0.351, 95% CI: −0.115–0.565).

**Conclusion:**

A time-dependent model using routinely obtained laboratory tests can accurately predict progression to CVCs in HD patients.

## Background

The global prevalence of end-stage renal disease (ESRD) has been steadily increasing (1, 2). The total population of individuals with ESRD receiving renal replacement therapy (RRT) surpassed 2.5 million in 2010 and is projected to exceed 5.439 million by 2030, with the majority of growth anticipated in Asia, where numbers are expected to increase from 0.968 million to approximately 2.162 million (3). The primary modality for RRT continues to be hemodialysis (HD), rather than peritoneal dialysis (PD) or kidney transplantation (4). The mortality rate in HD patients is high, primarily attributed to the occurrence of cardiocerebral vascular events (CVCs) (5–7). The clinical decision-making process for HD patients is challenging due to the diverse etiologies of kidney diseases, variable rates of disease progression, and competing risks of CVCs mortality. The accurate prediction of risk could enhance personalized decision-making, facilitating timely and appropriate patient care.

Currently, there is a lack of universally accepted predictive tools for the incidence of CVCs in HD patients. Therefore, physicians cannot identify patients at high risk of CVCs from HD patients in a timely manner or implement timely interventions to prevent or reduce the eventual occurrence of CVCs.

Recent studies have demonstrated that albuminuria (ALB) (8, 9) provides additional prognostic information regarding the progression to cardiovascular events (CVEs) in HD patients. One study investigated the incorporation of serum phosphate (P) and white blood cell (WBC) counts into prediction models (10). However, these models are either specific to CVEs but not CVCs or lack external validation. More accurate information regarding the risk of progression to CVCs in HD patients is required for clinical decisions about testing, treatment, and referral. The ideal model for predicting progression to CVCs in HD patients should have accuracy, be easy to implement, and demonstrate high generalizability across diverse patient populations in dependent cohorts.

Utilizing data extracted from the electronic medical records system (EMR) in three distinct HD centers, our study aimed to develop and externally validate an accurate but simple predictive model for the progression to CVCs in HD patients. The objective was also to utilize routinely measured variables in HD patients to develop a time-dependent predictive model for the progression of CVCs that could be easily implemented in clinical settings. We were particularly intrigued by models that exclusively rely on clinical laboratory data, facilitating the reporting of CVCs risk alongside laboratory test results.

## Materials and methods

### Study population

#### Development cohort

The development cohort was derived from the Kidney Disease Center at Dongguan Tungwah Hospital, Dongguan, Guangdong, China, and the Department of Nephrology at the Luogang Branch of the Third Affiliated Hospital of Sun Yat-sen University, Guangzhou, Guangdong, China, between 2017 and 2021. The patients underwent two to three weekly sessions of HD for a minimum duration of 3 months, employing bicarbonate-based dialysate and polysulfone membrane dialyzers. Low-molecular-weight heparin was administered as an anticoagulant during the dialysis procedure. The criteria for sample selection were as follows: patients who were at least 18 years old at the time of initiation of HD and who underwent quarterly biochemical tests and who had no history of prior renal transplantation or surgical procedures within the past year. Individuals with cachexia, malignant tumors, or other life-limiting conditions and patients who had experienced significant blood loss or severe infectious diseases during the study period were excluded from the study. The censored data were as follows: among the 56 screened patients who were excluded, reasons for exclusion included patient refusal to participate (*n* = 35), renal transplantation (*n* = 10), withdrawal of consent (*n* = 8), and poor adherence to HD treatment (*n* = 3).

#### Validation cohort

The validation cohort was derived from the Department of Nephrology at the Tianhe Branch of the Third Affiliated Hospital of Sun Yat-sen University, Guangzhou, Guangdong, China, between 2017 and 2021. The criteria for sample selection and exclusion were the same as those for the development cohort. The study was reviewed and approved by the institutional review boards at Dongguan Tungwah Hospital, Dongguan, Guangdong, China, and the Third Affiliated Hospital of Sun Yat-sen University, Guangzhou, Guangdong, China. Written informed consent was obtained from all patients or their immediate relatives, and the study was conducted in accordance with ethical guidelines (Ethical Batch No. 2020DHLL010).

#### Follow-up plan

The study investigated the occurrence of CVCs during a 5-year follow-up period. Upon occurrence of a cardiovascular event in a patient, we promptly initiated the consultation system and collaborated with the cardiologist to establish an accurate diagnosis of the cardiovascular event. In cases of cerebrovascular events, we sought consultation from a specialist in cerebrovascular medicine. We ensured that both diagnosing physicians held the title of attending physician or higher.

### Variables

#### Candidate independent variables

The candidate independent variables were selected based on their face validity, encompassing demographic factors such as age and sex, as well as physical examination variables, including blood pressure measurements before, during, and after the HD program, along with weight assessments; HD-related variables, including dialysis age and blood flow rate (BFR) during HD; and laboratory variables obtained from three different hemodialysis centers reflecting inflammation, nutrition and renal complications such as anemia (Table 1). The aforementioned data were collected on a regular quarterly basis. The baseline values were defined as the mean value within a 3-month period following enrollment. Exceptionally, the baseline values of serum creatinine (Cr) and blood urea nitrogen (BUN) were selected as the first values before HD after enrollment. Variables with more than 30% missing values were not included in the analysis.

**Table 1:** Baseline characteristics of the development and validation cohorts.

Characteristics	Development cohort (*n* = 233)	Validation cohort (*n* = 215)	*p* values
Demographics			
Age, mean (SD), y	56 (15)	53 (16)	0.26
≥65	75	64	NA
<65	158	151	NA
Male proportion (%)	63.52	56.74	0.14
Dialysis-related			
Dialysis age, mean (SD), m	47 (41)	9 (18)	0.087
Pre-HD SP, mean (SD), mmHg	149.82 (15.37)	153.06 (17.81)	0.29
Pre-HD DP, mean (SD), mmHg	81.47 (10.10)	82.91 (12.17)	0.23
Post-HD SP, mean (SD), mmHg	150.61 (19.05)	148.81 (18.80)	0.51
Post-HD DP, mean (SD), mmHg	85.31 (12.95)	83.11 (11.50)	0.39
Access for HD			
SCVC (%)	35.6	10.2	0.005
LCVC (%)	8.2	3.3	0.045
AVF (%)	51.5	85.1	0.003
AVG (%)	4.7	1.4	0.801
BFR, mean (SD), ml/min	208.25 (25.30)	216.46 (25.14)	0.043
UV, mean (SD), ml	1605.61 (1015.82)	2049.30 (1059.50)	0.902
Laboratory data			
HGB, mean (SD), g/L	102.81 (18.66)	100.50 (19.84)	0.22
HCT (%)	32.6 (6.9)	30.4 (5.9)	0.001
TSAT (%)	31.9 (15)	28.0 (15)	0.063
WBC, mean (SD), G/L	6.73 (2.67)	6.36 (1.94)	0.014
ALB, mean (SD), g/L	37.61 (4.48)	37.67 (3.67)	0.017
Cr, mean (SD), μmol/L	979.68 (369.75)	1041.56 (343.70)	0.461
BUN, mean (SD), mmol/L	25.28 (8.26)	28.67 (8.30)	0.006
CHOL, mean (SD), mmol/L	3.99 (0.94)	4.24 (1.09)	0.004
iPTH, mean (SD), pg./mL	467.82 (562.32)	512.11 (571.26)	0.205
K, mean (SD), mmol/L	4.96 (0.78)	4.94 (0.90)	0.005
Ca, mean (SD), mmol/L	2.17 (0.22)	2.13 (0.23)	0.008
P, mean (SD), mmol/L	2.00 (0.62)	2.15 (0.68)	0.012
Fe, mean (SD), μmol/L	12.92 (6.52)	10.64 (5.35)	0.038
Outcome			
Average time for CVCs, mean (SD), d	670.46 (529.32)	874.69 (650.28)	0.151
Outcome counts	74 (31.7%)	41 (19.0%)	NA

The patients in the development and validation cohorts had similar demographics and clinical variables. The incidence of CVCs was greater in the development cohort than in the validation cohort (31.7% vs 19.0%). *p* values less than 0.05 indicated a significant correlation between the occurrence of CVCs and the variables. HD, hemodialysis; CVCs, cardiocerebral vascular events; BFR, blood flow rate during HD; UV, ultrafiltration volume during HD; HGB, hemoglobin; HCT, hematocrit value; TSAT, transferrin saturation; WBC, white blood cell count; ALB, serum albumin; Cr, serum creatinine; BUN, blood urea nitrogen; CHOL, cholesterol; iPTH, intact parathyroid hormone; K, serum potassium; Ca, serum calcium; P, serum phosphorus; Fe, serum ferrium; SP, systolic pressure; DP, diastolic pressure; SCVC, short-term central venous catheter; LCVC, long-term central venous catheter; AVF, arteriovenous fistula; AVG, arteriovenous graft. Unless stated, the data are presented as the mean ± SD. The chi-square test was performed to clarify the correlation between the occurrence of CVCs and variables, and *p* values are shown. *p* < 0.05 was considered to indicate statistical significance.

#### Dependent variable

The outcome of interest was CVCs, including acute heart failure, acute hematencephalon, cardiac- or brain-derived death, acute myocardial infarction, acute cerebral infarction, ischemic cardiomyopathy, unstable angina pectoris, and stable angina pectoris. Upon the occurrence of a cardiovascular event in a patient, we promptly initiated the consultation system and collaborated with the cardiologist to establish an accurate diagnosis. For patients with cerebrovascular events, we sought consultation from a specialist in cerebrovascular medicine. We ensured that both diagnosing physicians held the designation of attending physicians or higher. The prediction of the risk of CVCs occurrence has significant implications for decision-making processes undertaken by patients, physicians, and healthcare systems. The time horizons for risk prediction were 1, 3, and 5 years.

### Statistical analysis

#### Model development

We developed a sequential series of models and compared them based on similar variable filtering conditions. We employed a combination of clinical guidelines and forward selection methodology to ascertain the selection of variables. Variables not significantly associated with CVCs (*p* > 0.10) were excluded from further analyses, except for Model 6, which was based on the chi-square test. Multiple testing adjustment: Bonferroni correction was applied to control type I error rate, with adjusted significance level at *α* = 0.002 (0.05/25 variables). The enhancement of model performance in time-dependent multivariate Cox proportional hazards regression models was evaluated by incorporating new candidate variables, with discrimination and goodness-of-fit metrics employed for assessment. Seven sequential models were developed with distinct variable selection criteria, Model 1: Variables with *p* < 0.005 in Cox regression; Model 2: *p* < 0.01; Model 3: *p* < 0.05; Model 4: *p* < 0.1; Model 5: All candidate variables; Model 6: Clinically meaningful variables reflecting anemia, nutrition, inflammation, and dialysis adequacy; Model 7: Variables with both clinical relevance and *p* < 0.1.

#### Time-varying covariates

Laboratory parameters (e.g., ALB, WBC) were updated at each hemodialysis session and analyzed as time-dependent covariates in extended Cox models.

#### Prediction model validation

By incorporating the concordance index (C index) and Akaike information criterion (AIC) and models 3, 4 and 6, the most accurate models among all the models were evaluated in the external validation dataset. The baseline hazard function and coefficients derived from the developed model were kept constant and applied to the validation dataset.

#### Prediction model performance

We employed a range of methodologies to assess the performance of the models across both the development and validation datasets.

##### Discrimination

Discrimination refers to the model’s ability to accurately differentiate between two classes of outcomes, namely, the occurrence and nonoccurrence of CVCs. The computation of the C index serves as a measure for assessing discrimination (11–13).

##### Calibration

The process of calibration refers to the extent to which the predicted probabilities align numerically with the observed outcomes. We compared the observed and predicted risks of CVCs occurrence across all time points and quantified the magnitude of deviation between them (14).

##### Goodness of fit

The overall model fit for sequential models was compared using the AIC, which incorporates both the statistical goodness of fit and the complexity of the model by penalizing an increase in parameters required to achieve a certain level of fit (11).

##### Reclassification

Reclassification refers to the reassignment of patients from one class to another based on changes in their risk category assignment. The improvement in reclassification was quantified using the net reclassification improvement (NRI) statistic (13).

#### Sensitivity analysis

The patients were categorized into subgroups based on the occurrence of endpoint events (CVCs). Two models from Model 3, Model 4, and Model 6 were selected to predict the endpoint events in these subgroups. A sensitivity analysis was conducted to evaluate the predictive efficacy of the models by comparing the positive or negative prediction probability with the actual positive or negative rate (predictions falling within a certain range can be considered accurate).

All the statistical analyses were performed using SPSS 20.0. A two-sided *p* < 0.05 was considered to indicate statistical significance.

## Results

### Cohort description

**Figure 1:**
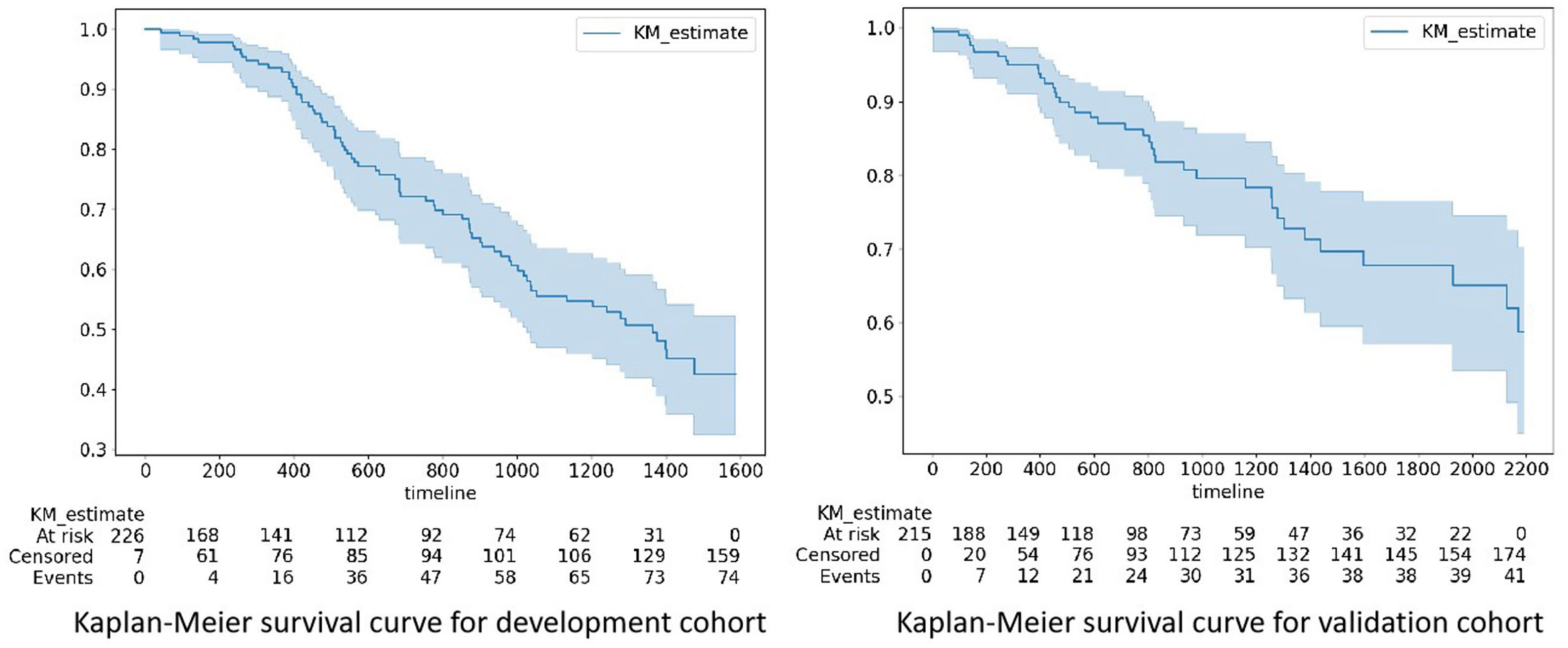
Kaplan–Meier survival curves for the development and validation cohorts. The proportion of events was greater in the development cohort than in the validation cohort (74 patients with CVCs occurrence [31.7%] vs. 41 patients with CVCs occurrence [19.0%]). CVCs, cardiocerebral vascular events. At risk: the number of patients with potential risks at a specific time point; Censored: the number of patients censored at a specific time point; Event: the cumulative count of risk events that transpired until a specific time point.

The development and validation cohorts included 233 and 215 patients, respectively (Table 1). Patients in the development and validation cohorts were similar in age and sex. The HD-related variables pre- and post-HD blood pressure, BFR, and UV were similar between the two cohorts. Laboratory data, such as hemoglobin (HGB), WBC, ALB and so on, were similar between these two cohorts. The proportion of events was greater in the development cohort than in the validation cohort (74 patients with CVCs occurrence [31.7%] vs. 41 patients with CVCs occurrence [19.0%]) (Figure 1).

A chi-square test was performed on all patients to clarify the correlation between the occurrence of CVCs and variables, and the *p* values are shown in Table 1. *p* values less than 0.05 indicated a significant correlation between them. Indicators were chosen for the prediction model based on statistical correlation and clinical practice.

### Prediction model performance in the development cohort

The hazard ratios for the variables and statistics regarding discrimination and goodness of fit for successive models in the development dataset are presented in Table 2. According to the chi-square test presented in Table 1, variables that showed no significant association with CVCs (*p* > 0.10) were excluded from further analyses, except for Model 6.

**Table 2:** Hazard ratios and goodness of fit for sequential models in the development dataset.

Variables	Models						
	1	2	3	4	5	6	7
Age					0.05	<0.005	
Male sex					0.01	0.14	
Pre-HD SP					0.57		
Pre-HD DP					0.12		
Post-HD SP					0.54		
Post-HD DP					0.97		
SCVC (%)	0.67	0.38	0.76	0.75	0.90		
LCVC (%)			0.15	0.21	0.22		
AVF (%)	0.28	0.35	0.51	0.51	0.33		
AVG (%)					0.46		
BFR			0.22	0.31	0.31	0.67	0.67
UV					0.47	0.58	
HGB					0.01	0.25	
HCT	0.11	0.03	0.31	0.35	0.20		0.99
TSAT				0.76	0.45		
WBC			0.09	0.10	0.04	0.19	0.15
ALB			<0.005	<0.005	0.01	0.03	<0.005
Cr					0.45		
BUN		0.72	0.65	0.55	0.17		0.74
CHOL	0.40	0.34	0.07	0.10	0.05		
iPTH					0.53		
K	0.56	0.93	0.75	0.7	0.81		0.90
Ca		0.67	0.36	0.5	0.49		
P			0.66	0.63	0.41	0.23	0.60
Fe			0.79	0.78	0.67		
C index	0.596	0.654	0.718	0.725	0.772	0.704	0.68
95% CI	0.523–0.668	0.585–0.722	0.649–0.786	0.658–0.791	0.709–0.834	0.639–0.768	0.613–0.746
AIC	683.92	677.19	664.2	666.72	659.61	659.22	669.81
*p* value	<0.001	<0.001	<0.001	<0.001	<0.001	<0.001	<0.001

The units of the variables are consistent with those presented in Table 1. Higher values for the C index and lower values for the AIC indicate better models. By incorporating the C-index and AIC, models 3, 4 and 6 were chosen for evaluation in the external validation dataset. HD, hemodialysis; BFR, blood flow rate during HD; UV, ultrafiltration volume during HD; HGB, hemoglobin; HCT, hematocrit value; TSAT, transferrin saturation; WBC, white blood cell count; ALB, serum albumin; Cr, serum creatinine; BUN, blood urea nitrogen; CHOL, cholesterol; iPTH, intact parathyroid hormone; K, serum potassium; Ca, serum calcium; P, serum phosphorus; Fe, serum ferrium; SP, systolic pressure; DP, diastolic pressure; SCVC, short-term central venous catheter; LCVC, long-term central venous catheter; AVF, arteriovenous fistula; AVG, arteriovenous graft. *p* < 0.05 was considered to indicate statistical significance.

Model 1, which included serum potassium (K), cholesterol (CHOL), hematocrit (HCT) and HD vascular access types (accesses), performed poorly (C index, 0.596; 95% confidence interval [CI], 0.523–0.668). The C index improved with the inclusion of serum calcium (Ca), BUN and blood glucose (GLU) in Model 2 (0.654; 95% CI, 0.585–0.722). The addition of more indicators from Model 1 to Model 5 led to a gradual increase in the C index value. Model 6 and Model 7, each containing representative clinical variables, which reflect the state of anemia, nutrition and inflammation, electrolyte levels and dialysis adequacy, showed similar performances (C index 0.704 for Model 6; C index 0.680 for Model 7). Despite a similar C index, the AIC was lower for Model 6 than for Model 7 (659.2 vs. 669.8, respectively). The inclusion of all variables in Model 7 had the largest C index (0.772; 95% CI, 0.709–0.834) among all models, and the AIC was lowest for Model 6 (AIC: 659.2) compared with the other models. Given these results, models 1, 2, and 7 were not considered in further evaluation steps.

### Performance of the prediction model in the validation cohort

**Figure 2:**
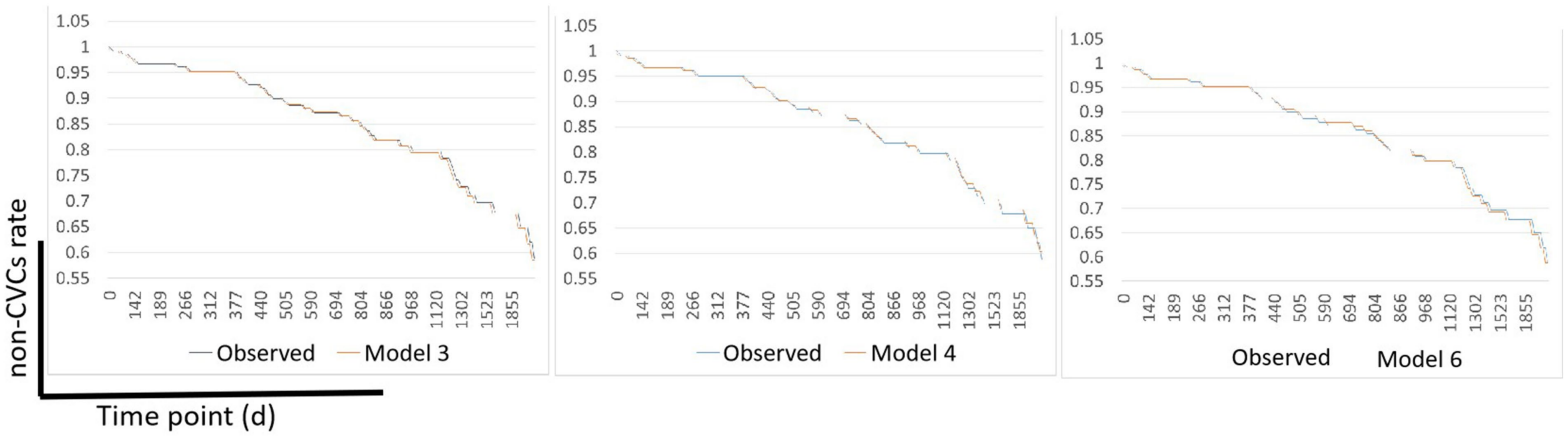
Observed vs. predicted non-CVCs rates using Models 3, 4, and 6 in the validation cohort. The MSE difference between the observed and predicted probabilities over the entire time-point of the CVCs was lower for Model 6 than for Models 3 and Model 4 (0.0067, 0.0059, and 0.0052, respectively). MSE, mean square error; CVCs, cardiocerebral vascular events.

After screening by the C-index and AIC, Model 3, Model 4, and Model 6 were selected to determine their ability to predict CVCs occurrence in the validation cohort. Survival was calculated using time-dependent Cox regression and compared with the observed Kaplan–Meier (KM) curve of the validation cohort. Figure 2 shows the observed vs. predicted non-CVCs rates at all time points for models 3, 4, and 6 in the validation cohort. The mean square error (MSE) difference between the observed and predicted probabilities over the entire period of risk was lower for Model 6 than for Models 3 and 4 (0.0067, 0.0059, and 0.0052, respectively).

**Figure 3:**
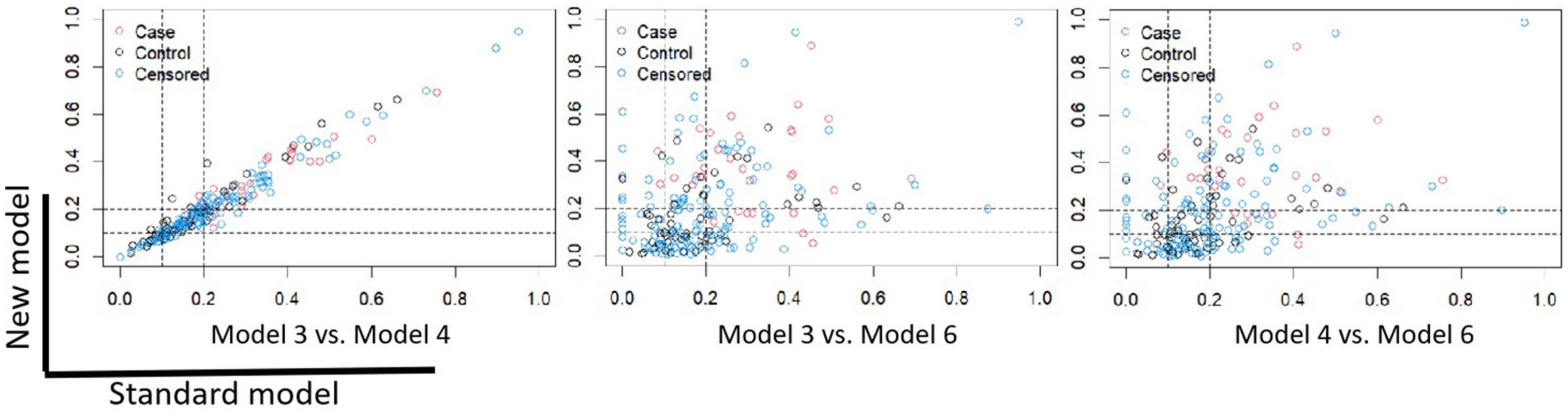
Scatter plot of the NRIs for Model 3, Model 4, and Model 6. The 1200th day time node was chosen for NRI analysis. Model 3 vs. Model 4: NRI -0.007 (95% CI: −0.220, 0.246); Model 3 vs. Model 6: NRI 0.351 (95% CI: −0.115, 0.565); Model 4 vs. Model 6: NRI 0.329 (95% CI: −0.085, 0.604). NRI, net reclassification improvement.

The NRI risk for CVCs (Figure 3) in the validation cohort was analyzed. The 1200th daytime node was chosen for NRI analysis. Overall, model 6 outperformed models 3 and 4, with NRIs of 0.351 (95% CI, −0.115–0.565) and 0.329 (95% CI, −0.085–0.604), respectively.

### Sensitivity analyses

Sensitivity analyses of each model were performed by comparing the difference in the prediction of positive and negative events for the validation cohort. The accuracy of predicting the occurrence of positive events, specifically CVCs, was represented by the NRI + (positive net reclassification improvement), as demonstrated in Table 3. Conversely, the NRI represents the accuracy of predicting the absence of CVCs.

**Table 3:** Sensitivity analyses for Model 3, Model 4, and Model 6.

Models	NRI	NRI+	NRI−
3 vs. 4	−0.007 (−0.220, 0.246)	−0.047 (−0.244, 0.114)	0.004 (−0.220, 0.246)
3 vs. 6	0.351 (−0.115, 0.565)	0.131 (−0.084, 0.387)	0.220 (−0.058, 0.387)
4 vs. 6	0.329 (−0.085, 0.604)	0.155 (−0.005, 0.368)	0.173 (−0.137, 0.341)

The NRI+ represents the accuracy in predicting the occurrence of CVCs. The NRI- represents accuracy in predicting the absence of CVCs. The predictive performance of Model 6 surpassed that of Model 3 and Model 4 in determining the occurrence of CVCs or the absence of CVCs in HD patients. NRI, net reclassification improvement; CVCs, cardiocerebral vascular events.

The predictive performance of Model 6 surpassed that of Models 3 and 4 in determining the occurrence of CVCs in HD patients (Model 3 vs. Model 6: 0.131, 95% CI, −0.084–0.387; Model 4 vs. Model 6: 0.155, 95% CI, −0.005–0.368). Additionally, model 6 outperformed models 3 and 4 in predicting the absence of CVCs in HD patients (model 3 vs. model 6: 0.220, 95% CI, −0.058–0.396; model 4 vs. model 6: 0.173, 95% CI, −0.137–0.341).

### Correlation coefficients and HR values for model 6

Correlation Coefficients and HR Values for Model 6 are presented in Table 4. These data represent the coefficients of the time-dependent Cox regression model, where positive and negative signs indicate positive and negative correlations with the endpoint event, respectively. The HR values quantify the extent of influence on the endpoint event. The PH test indicated that all independent variables, except for gender, satisfy the assumption of proportional hazards. Therefore, the correlation coefficient of gender showed as a time-dependent covariate coefficient, while HR represents a dynamic value that varies over time.

**Table 4:** Correlation coefficients and HR values for Model 6.

Model 6	Age	BFR	UV	HGB	ALB	WBC	P	Male*	T_cov_Male*
Correlation coefficients	0.028	−0.002	−0.001	−0.009	−0.080	0.045	0.341	0.419	0.413
HR values	1.029	0.998	0.999	0.991	0.923	1.046	1.406	exp [0.419 + 0.413*ln(days)]	

The coefficients’ positive and negative signs indicate a positive and negative correlation with the endpoint event, respectively. *Male gender, The PH test revealed that the assumption of proportional hazards was not satisfied by the male gender. BFR, blood flow rate during HD; UV, ultrafiltration volume during HD; HGB, hemoglobin; WBC, white blood cell count; ALB, serum albumin; P, serum phosphorus.

## Discussion

In patients who initiate HD, the early mortality rate is high (15), with up to 40% of deaths attributed to cardiovascular disease (16, 17). Among patients undergoing maintenance HD, cardiovascular incidents continue to be the primary cause of mortality (18). Moreover, the incidence of cerebrovascular events is significantly greater in HD patients than in the general population (6, 7). Despite this knowledge, the risk factors for CVCs in HD patients have yet to be fully elucidated to establish a practical risk prediction model. However, insufficient research has investigated cardiovascular and cerebrovascular events in HD patients.

Risk prediction has garnered increasing attention in the past two decades, with the emerging literature suggesting enhanced patient outcomes through personalized risk prediction and advancements in information technology facilitating seamless integration of risk prediction models into electronic health records (EHRs) (19–22). The availability of these risk prediction tools and their incorporation into clinical practice guidelines has resulted in enhanced adherence to treatment guidelines and facilitated individual decision-making (23, 24). Despite these advantages, the lack of readily applicable and externally validated models has impeded the widespread integration of risk prediction across all medical disciplines (25).

We developed and validated a set of time-dependent risk prediction models for progression to CVCs among patients undergoing HD. Our models utilize laboratory data routinely obtained from patients undergoing HD, which can be easily integrated into a laboratory information system or clinic EHRs.

Among patients undergoing HD, there is significant heterogeneity in the risk of progressing to CVCs. For instance, Table 5 illustrates the predicted probability of CVCs occurrence in two real patients with similar clinical conditions using models 3, 4, and 6. For patient A, with an observed time of 3.17 years for CVCs, models 3 and 4 exhibited similar predictive probabilities at the 3-year time point, while model 6 demonstrated an obviously elevated value. Model 6 provides substantially different risk predictions than models 3 and 4. In comparison to Model 3, Model 6 demonstrated a 28% increase in the predicted risk for Patient A at the 3-year time point and an 18% increase in the risk for Patient B at the 1-year time point.

**Table 5:** Predicted probability of CVCs for 2 patients using our prediction models^a^.

Model	Dialysis age	Probability of CVCs, %	
		Patient A (72-year-old male, with the observed time for CVCs 3.17 years)	Patient B (79-year-old male, with the observed time for CVCs 1.15 years)
3	1 year	3	8
	3 years	14	32
	5 years	25	51
4	1 year	3	5
	3 years	11	21
	5 years	19	35
6	1 year	10	26
	3 years	42	79
	5 years	65	96

^a^The risk calculator can be accessed in Supplementary material 1. NRI, net reclassification improvement; CVCs, cardiocerebral vascular events.

Our models rely on demographic data and laboratory markers reflecting dialysis adequacy or complications associated with chronic kidney disease (CKD) to predict the future risk of CVCs occurrence in HD patients. Similar to previous findings from Xiaobing Liu (26) and Yukiko Matsubara (8), we observed that lower levels of HGB, ALB, UV, and BFR, as well as male sex, were associated with faster progression to CVCs. The time-varying HR for male gender (HR = exp.[0.419 + 0.413 × ln(t)]) suggests accumulating risk over dialysis vintage. This pattern may be attributed to the vascular calcification process and fluctuations in hormone levels, both of which warrant further research. Furthermore, higher WBC and serum P levels, along with older age, were also predictive of an increased risk of CVCs in HD patients. These markers may reflect dialysis adequacy or underlying processes of inflammation or malnutrition.

In contrast to previous studies that have individually associated these laboratory markers with the progression to CVCs, our research integrates them collectively into a unified risk equation (the risk calculator can be accessed in Supplementary material 1). In addition, we demonstrated no improvement in model performance with the addition of vascular access variables and physical examination (systolic blood pressure, diastolic blood pressure). Although these variables are clearly important for the diagnosis and management of HD patients, the lack of improvement in model performance may reflect their broad applicability.

Our study has several limitations. First, the inclusion of three independent dialysis centers in large general hospitals restricts the generalizability of our findings to patients undergoing HD in community hospitals or community healthcare centers. Second, it should be noted that the majority of participants in this study were of Han nationality, which reflects the ethnic distribution within Guangdong Province, China. However, it is important to acknowledge that certain underrepresented ethnic minorities may have a greater susceptibility to CVCs. Third, the sample size in this study was relatively limited, which may have influenced the stability of the model due to the low incidence of CVCs events. Future research will involve a multicenter study enrolling at least 2000 patients to validate these findings.

In summary, we have developed and validated time dependent, highly precise predictive models for progression to CVCs in patients undergoing HD. The optimal model we developed utilizes readily available laboratory data and demonstrates high accuracy in predicting the short-term risk of CVC occurrence. Moreover, its seamless integration into a laboratory information system ensures easy implementation. Model parameters can be integrated into hospital electronic health record systems to automatically compute risk scores and trigger alerts following each dialysis session. Prospective studies are currently being planned to assess the impact of the model on clinical decision-making, including modifications to dialysis regimens or enhanced cardiovascular monitoring protocols. Furthermore, future research will focus on optimizing the model’s performance through systematic exploration. External validation across multiple diverse cohorts of HD patients and evaluation in clinical trials are imperative.

## Data Availability

The raw data supporting the conclusions of this article will be made available by the authors, without undue reservation.
